# Depression, Anxiety and Quality of Life in Long-Term Survivors of Malignant Melanoma: A Register-Based Cohort Study

**DOI:** 10.1371/journal.pone.0116440

**Published:** 2015-01-23

**Authors:** Manfred E. Beutel, Sabine Fischbeck, Harald Binder, Maria Blettner, Elmar Brähler, Katharina Emrich, Peter Friedrich-Mai, Barbara H. Imruck, Veronika Weyer, Sylke R. Zeissig

**Affiliations:** 1 Department for Psychosomatic Medicine and Psychotherapy, University Medical Center Mainz, Mainz, Germany; 2 Institute for Medical Biostatistics, Epidemiology and Informatics (IMBEI), University Medical Center Mainz, Mainz, Germany; 3 Medical Psychology and Sociology, Department for Psychosomatic Medicine and Psychotherapy, University Medical Center Mainz, Mainz, Germany; Iranian Institute for Health Sciences Research, ACECR, ISLAMIC REPUBLIC OF IRAN

## Abstract

**Aim:**

The purpose of the study was to determine anxiety and depression, quality of life, and their determinants in long-term survivors of malignant melanoma.

**Methods:**

In a state cancer registry a cohort of survivors of malignant melanoma was contacted via the physician registered. Of 1302 contactable patients, 689 (52.2%) completed a questionnaire including the Patient Health Questionnaire with generalized anxiety (GAD-7) and depression (PHQ-9) and the EORTC Quality of Life Questionnaire (EORTC QLQ 30). Based on multiple regression analysis, predictors of quality of life and distress were identified. Comparison data were assessed in two waves of representative face-to-face household surveys of the adult German population.

**Results:**

An average of 8.4 (5.7 to 12.2) years after diagnosis, distress was higher in women compared to men and in middle adulthood (vs. older patients). Symptoms were higher in women than in men, and there was a decline of functioning and increase of symptoms across the age range of both genders. Compared to the general population, there were slightly increased depression and anxiety (only women), but no impaired global quality of life. Yet, survivors evidenced functional decline and more physical symptoms. Distress and reduced quality of life were consistently predicted by lack of social support, fear of recurrence, pessimism and self-blame. Distress was increased by a family history of melanoma, and additional mental and somatic diseases.

**Conclusion:**

Overall, long-term survivors have adjusted well achieving a global quality of life comparable to the general population. Yet, compromised functional dimensions, physical symptoms and distress indicate the need for integrating psychooncological screening into oncological follow-up, which might be guided by predictors such as family history or social support. Further prospective study is needed to determine the course of adaptation to the disease and corroborate the risk factors identified.

## Introduction

Due to its rapid spread in the population, malignant melanoma has become one of the most frequent forms of cancer, affecting up to 22.2 per 100,000 population in the USA [1]. In a systematic review [2] on average about 30% of patients with malignant melanoma suffered from heightened distress around the time of diagnosis and treatment. Between 18 and 44% of participants scored in the clinical range for anxiety (based on the HADS) (depressive symptoms 6 to 28%). Recently [3], we found in a consecutive series of 520 melanoma patients that 47% reported heightened distress (distress thermometer score ≥5) regarding emotional sources of distress, practical and work problems, family, partner, and physical problems (like pain, appearance, getting around, and nausea).

Identified risk factors for heightened distress were female sex [2, 4, 5], younger age [2–4], lower education [2], a lack of partnership and social support [2, 5], and a negative appraisal of disease and coping ability [2, 4, 5, 6, 7]. Distress was heightened by physical deterioration, visibility of the body site affected [2] and current systemic treatment [2, 3].

Detected at an early stage, the vast majority of cases are treated effectively. Still, survivors of malignant melanoma have to grapple with ongoing threat of recurrence and requirements of reduction of UV exposure, continued self-examination and dermatological controls. While regular self- and clinical skin examination may provide a sense of control, survivors of malignant melanoma have also described an enduring fear of developing a new melanoma [4]. Research to date has tended to focus on identifying risk factors and the level if distress around the time of diagnosis. Thus, it remains unclear, to what extent long-term survivors’ quality of life is compromised and if they experience lasting psychological distress. In a single center study, Stava et al. [8] compared survivors of melanoma (996) to other forms of cancer (N = 7563) about 19 years after diagnosis. Overall, melanoma survivors reported their health less frequently as affected in the long run (15.8%) compared to the other types of cancer (34.9%). In an Australian study focusing on physical aspects, melanoma long-term survivors more frequently suffered from somatic comorbidity, worse overall health and well-being compared to the general population [9]. Only few long-term registry-based studies have been performed on malignant melanoma to date [10]. A Dutch registry-based follow-up study of 562 former melanoma patients found quality of life (SF-36) up to 9 years after diagnosis comparable to the general population; time since diagnosis, tumor stage and comorbidity were predictors of tumor-related concerns [11].

International medical guidelines aiming at early detection of recurrence have not yet arrived at a uniform approach [12]. While meant as a measure of reassurance, follow-up examinations may also remind patients of past threat and activate fears of recurrence or new disease [4]. Yet, medical guidelines have failed to specify recommendations for screening for distress or wish for support in the medical aftercare of melanoma patients [13]. Thus, as patients have indicated in surveys, little attention has been paid to their emotional well-being in medical follow-up [13, 14]. Better knowledge about long-term psychosocial strains and related risk factors is needed in order to inform medical and psychooncological aftercare [14]. The purposes of this population-based study were therefore to determine the level and determinants of (1) quality of life and (2) anxiety and depression in long-term survivors of malignant melanoma.

## Materials and Methods

### Participants

In the state of Rhineland-Palatinate, all cancer patients are registered by their physicians in a cancer registry, at the University Medical Center, Mainz. Data include personal identification in a pseudonymous form, and tumor diagnosis (ICD 10), topography and morphology (ICD-O-3), staging (TNM), age, sex and date of diagnosis. Estimated completeness of incident melanoma notifications in Rhineland-Palatinate is more than 95%. We included all registered former patients with a diagnosis of malignant melanoma (ICD-10: C43) registered by their dermatologist from 2000 to 2005 a) alive at the assessment, b) aged at least 14 years at diagnosis, c) who gave their written informed consent. For legal reasons and confidentiality, the cancer registry decoded the patients’ names. The physician who had registered contacted the patient and forwarded study information, written informed consent form and questionnaire. Coded data were analyzed without reference to personal identification. Patients who did not react to the letter of interest within six weeks got a reminder letter. Non-Responders were not contacted further. The protocol was approved by the Ethics Committee of the Statutory Physician Board of the State of Rhineland Palatinate (Reference number 837.161.11 (7703)).

### Measures and potential predictors

Depression, anxiety and global quality of life were our primary outcome measures. Depression was measured by the Patient Health Questionnaire (PHQ) depression module (PHQ-9) [15], anxiety was assessed by the General Anxiety Disorder Questionnaire (GAD-7) of the PHQ [16]. We used the mean score of global health status and quality of life of the Quality of Life Core Questionnaire EORTC-QLQ-C30 [17] to measure global quality of life. As secondary outcomes we assessed the five functional scales, three symptom scales, and six single items assessing common symptoms and the perceived financial impact of the disease. Scales were transformed to a 0–100 scale. Higher functional and global health scores represent better functioning, whereas higher symptom scores reflect greater symptom distress.

The following variables were used as predictors of distress and quality of life: The 24-item Illness-specific Social Support Scale (ISSS) assesses positive social support (15 items) and detrimental interaction (9 items) based on 5-point Likert scales [18]. The Brief Cope (BC) [19] consists of 14 coping scales, each with two items. We used three factor-analytically derived scales with acceptable reliability (accounting for 38% of total variance): “Seeking external support” (Cronbach α =. 75), “Denial/Self-blame” (α = .74), and “Active coping” (α = .76). The Life-Orientation-Test (LOT) reliably [20] assesses generalised optimism and pessimism (10 items). Fear of recurrence was assessed by one item from the Hornheide Questionnaire (HQ-S) [21]. Social and medical characteristics were also assessed.

### Comparison groups

To obtain comparison data, generalized anxiety (GAD-7) [16], Quality of life (EORTC QLQ30) [22] and depression (PHQ 9) [23] were assessed in two waves of representative face-to-face household surveys of the German population (age range 14 to 94 years); study participants were presented the questionnaires by professional interviewers of a demographic consultation company (USUMA, Berlin). In the 2007 survey, a total of 5036 (62.1% of all eligible) persons agreed to participate, provided verbal informed consent, and completed the GAD-7. Considering the age distribution of the former patients, only subjects at the age of 30 years or older were analyzed. Thus, the reference sample consisted of 4133 subjects at age 30 and older with 53.7% females. Sample sizes in the age classes were: 30–39 years: 19.4%; 40–49: 22.8%; 50–59: 19.8%; 60–69: 21.7%; 70+: 16.3%. The EORTC QLQ30 and the PHQ-9 were completed by 2515 participants with comparable response rates (57.7%) at the time of the cancer survey (2011 to 2012). The reference population for EORTC QLQ30 and PHQ9 (at least 30 years) consisted of 2049 participants (53.4% female; 30–39 years: 16.7%; 40–49: 19.3%; 50–59: 24.4%; 60–69: 20.3%; 70+: 19.3%).

### Statistical Analysis

To identify predictors of quality of life and distress linear regression models were used. Multiple imputation was performed to deal with missing values in covariates, resulting in several imputation data sets. 16 Regression models were first fitted separately for single imputation data set. Variable selection was performed by forward and backward selection with a selection level of 5% on two randomly chosen single imputation data sets. We selected a set of covariates with a p-value <.05 in at least one forward/ backward selection, fitted a regression model with the selected covariates for each single imputation data set, and combined the results by the SAS procedure Proc MIANALYZE. As a sensitivity analysis, for each criterion, a complete case analysis was performed excluding all persons with missing values. For highly skewed variables a log+1 transformation was performed prior to regression analysis. The level of significance was set to 5% and controlled by Bonferroni correction within the analysis for each outcome. All analyses were performed with SPSS 19, 21, respectively, SAS for Windows 9.2 TS Level 1M0 (SAS Institute Inc.) Cary, NC, USA.

Comparisons of quality of life and distress scores to the general population were performed separately for men and for women. Age groups were divided according to “30 to 39”, “40 to 49”, “50 to 59”, “60 to 69” and “70 and more” years. As older age groups were overrepresented in the melanoma sample compared to the general population, mean scores for men and women, were age-adjusted by multiple regression analysis. All further analyses were regarded as exploratory.

## Results

### Participant flow, recruitment

Out of 112 dermatologists who had registered 2113 patients between 2000 and 2005, 75 physicians participated (67%). Non-participating physicians (n = 37) reported no interest in studies, lack of time, no more personal patient contact or medical practice. Participating physicians had registered 1702 (80.5%) of the total patients who were therefore considered contactable. 382 patients could not be reached as being unavailable (200/ 11.8%), had died (46/ 2.7%) or were excluded by their physician (136/ 8%). Overall, out of 1320 contacted patients 689 (52.2%) entered into the statistical analysis.

Only 6 participants (.9%) were under the age of 30 years; 30–39 (6.7%); 40–49 (16.3%); 50–59 (19.7%); 60–69 (19.7%); 70+ (36.7%). Men (N = 335) and women (N = 354) were almost equally represented; 75.5% were married, and 82.7% lived in a partnership. 48.3% had primary, 28.4% 10^th^ grade, and 20.6% high school education. The majority (72%) lived in rural areas. The average time since diagnosis was 8.41 (SD = 1.72) years (range 5.67 to 12.17 years). According to the registry, the tumor stage at diagnosis (UICC) was 1 in 53%, 2 in 4.9% and 3 in 1.0%. A total of 41.1% of tumor stages were missing, however. Missing tumor staging resulted from the fact that in many instances the dermatologist registered the case before the presence of metastases could be excluded based on intensive diagnostic work-up. Based on the long follow-up period, it can be surmised that these were usually stage 1 cases. Recurrent disease was reported by 2.2% of the patients, metastases by 1.2%. 14.3% reported melanoma among close relatives (parents 7.4%, siblings 4.7%, children 2.4%). 5% reported the diagnosis of prior mental disease. A total of 78% suffered from additional chronic diseases (hypertension, thyroid disease, sleeping disorder, allergy, coronary heart disease, diabetes, other cancer.

### Depression, anxiety and quality of life in survivors of malignant melanoma

Table 1 presents depression and anxiety scores for former patients and for the general population, according to sex and age group.

**Table 1:** Depression (PHQ-9) and anxiety (GAD-7) in male and female malignant melanoma survivors and in the general population.

	**PHQ-9**						**GAD-7**					
**Age group**	**Melanoma**			**General Population**			**Melanoma**			**General population**		
	**All ^1)^ (N = 683)**	**Men (N = 333)**	**Women (N = 350)**	**All (N = 2049)**	**Men (N = 955)**	**Women (N = 1094)**	**All ^2)^ (N = 683)**	**Men (N = 333)**	**Women (N = 350)**	**All (N = 4133)**	**Men (N = 1914)**	**Women (N = 2219)**
Total	3.87(4.06)	3.45 (3.83)	4.37(4.25)	2.38(3.23)	2.29(3.27)	2.47(3.19)	3.33(3.77)	2.79(3.54)^3)^	3.86(3.93)	2.99(3.41)	2.70(3.25)	3.23(3.51)
30–39	4.48(4.42)	4.56 (4.13)	4.43(4.65)	1.60 2.62)	1.37(2.71)	1.79(2.54)	3.93 (3.76)1)	3.81 (3.80)	4.00(3.81)	2.84(3.51)	2.30(2.89)	3.23(3.85)
40–49	4.48(3.97)	3.81 (4.56)	4.70(3.76)	2.22(3.20)	2.07(3.45)	2.33(2.99)	3.93 (3.87)	3.12 (4.46)	4.19(3.65)	2.98(3.31)	2.68(3.14)	3.22(3.42)
50–59	4.82(4.71)	4.96 (4.81)	4.72(4.67)	2.35(3.25)	2.34(3.26)	2.36(3.24)	4.22 (4.14)	4.27 (4.41)	4.18(3.96)	3.13(3.46)	3.10(3.41)	3.16(3.51)
60–69	3.33(4.10)	2.97 (3.73)	3.86(4.58)	2.26(2.81)	2.01(2.60)	2.50(3.83)	3.25 (4.14)	2.72 (3.76)	3.98(4.53)	2.85(3.38)	2.65(3.41)	3.05(3.35)
70+	3.19(3.37)	2.87 (3.07)	3.78(3.80)	3.40(3.82)	3.52(3.82)	3.30(3.83)	2.44 (3.04)	2.09 (2.52)	3.09(3.74)	3.17(3.39)	2.69(3.29)	3.52(3.42)

^1)^Multiple regression, separately for men and women with group (melanoma vs. general population) as predictor: men: beta = 1.05 (95% CI: .59 to 1.51); p<.0001; women: beta = 1.87 (95% CI: 1.42 to 2.32); p<.0001

^2)^Multiple regression, separately for men and women with group (melanoma vs. general population) as predictor: men: beta = .13 (95% CI-.28 to .54); n.s.; women: beta = .59 (95% CI .17 1.02); p = .006

Considering only the former melanoma patients, both mean depression and anxiety scores were higher in women compared to men. When comparing the scores across the age range, depression and anxiety were lowest in men and women aged 70+. The highest scores were found in middle adulthood (aged 50–59 years). Mean depression scores were clearly below the clinical range (10 or higher).

Table 2 shows quality of life scores of patients, again separately for sex and age groups.

**Table 2:** Quality of life (EORTC QLQ30) in male and female survivors of malignant melanoma across the age range.

	Men						Women					
Age (years) ***Functional scales***	All N = 330–334 ^1); 2)^	30–39 N = 16	40–49 N = 28	50–59 N = 56–57	60–69 N = 77–79	≥70 N = 152–153	All N = 345–351	30–39 N = 30	40–49 N = 84	50–59 N = 77–78	60–69 N = 57	≥70 N = 93–98
Physical	86.1(19.7)	97.1(4.9)	95.2(11.2)	89.9(16.8)	90.0(16.9)	79.7(22.2)	86.6(19.3)	95.8(10.4)	95.2(10.3)	92.3(12.2)	86.7(17.0)	71.8(24.5)
Role	82.8(26.7)	95.8(11.4)	94.1(15.9)	84.8(24.7)	85.1(24.6)	77.6(30.0)	84.7(25.6)	91.1(18.4)	90.9(18.6)	88.2(21.5)	85.4(26.2)	76.2(27.5)
Emotional	78.3(24.0)	73.4(26.9)	78.3(26.5)	67.4(27.9)	81.9(22.4)	81.2(21.4)	71.4(25.1)	74.2(21.7)	69.8(24.3)	68.3(27.9)	73.8(27.5)	74.2(22.1)
Cognitive	81.9(22.0)	84.4(19.7)	87.5(21.6)	80.7(25.6)	94.2(12.8)	79.1(21.2)	82.2(22.9)	90.0(16.1)	84.7(20.6)	79.9(23.8)	82.2(26.0)	80.78(2.3)
Social	86.1(22.7)	89.6(19.1)	89.3(20.9)	85.1(24.6)	88.1(22.9)	84.4(22.7)	86.2(25.5)	92.8(15.6)	89.5(21.9)	84.8(26.2)	86.8(26.9)	82.7(29.1)
***Symptom scales***	
Fatigue	24.5(23.7)	20.1(20.4)	15.5(19.2)	27.7(25.2)	17.0(19.9)	29.0(24.9)	27.8(26.7)	23.7(23.5)	23.0(21.9)	24.6(25.3)	24.9(25.1)	36.5(30.6)
Nausea	2.3 (8.4)	1.0 (4.2)	1.8 (6.9)	4.2 (9.1)	2.2 (11.9)	1.8 (7.5)	4.0 (10.9)	3.9 (8.4)	3.0 (7.8)	5.3 (11.6)	3.8 (10.9)	3.7 (13.0)
Pain	22.2(28.4)	10.4(16.0)	8.3(16.0)	23.1(28.5)	20.3(27.2)	26.7(30.8)	23.6(31.3)	10.0(21.3)	17.7(26.8)	17.9(27.1)	25.7(30.7)	36.1(36.2)
Dyspnoea	15.8(27.1)	0	1.2(6.3)	15.2(23.6)	15.4(25.6)	20.8(31.1)	14.9(26.3)	3.3(13.4)	7.5(16.7)	10.6(20.4)	17.0(29.0)	28.0(33.8)
Insomnia	27.8(31.0)	31.3(31.0)	23.8(31.2)	29.8(30.9)	28.2(33.2)	27.2 (30.1)	33.6 (34.0)	18.9(25.8)	30.2(30.5)	36.3(37.4)	34.5(35.1)	39.3(35.1)
Appetite loss	3.6 (11.6)	4.2 (11.4)	2.4 (8.7)	6.7 (17.5)	2.1 (8.4)	3.3 (10.7)	6.3 (17.6)	5.6(12.6)	3.6(12.7)	6.4(19.4)	2.9(11.4)	10.5(22.8)
Constipation	6.2 (17.8)	0	2.4 (12.6)	1.8 (10.0)	2.2 (11.3)	11.2 (22.7)	11.5 (24.7)	5.6 (15.4)	7.6 (17.5)	7.7 (18.6)	11.1(23.0)	19.4 (5.6)
Diarrhoea	9.2 (20.8)	8.3 (14.9)	3.6 (10.5)	15.2(27.1)	8.2 (20.5)	8.8 (20.3)	8.6 (19.4)	6.7 (16.1)	6.0 (14.8)	9.8 (18.7)	10.1(24.6)	8.6 (19.6)
FinancialDifficulties	10.5 (24.4)	14.6(27.1)	9.5 (23.8)	14.3(29.7)	12.1(26.4)	8.1 (21.0)	8.4 (21.8)	3.3 (10.2)	6.4 (20.4)	10.3(25.4)	8.3 (22.3)	9.5 (20.9)
**Global**	71.1 (21.2)	75.5(15.1)	80.1(20.0)	68.8 (22.1)	74.5 (21.3)	68.0 (21.1)	70.0 (22.4)	79.7(21.6)	77.2(17.4)	72.2 (20.8)	70.0(24.1)	65.6(22.6)

^1)^Survivors below 30 years excluded due to small sample sizes (male: N = 2, female: N = 4);

^2)^ Means/SD are presented

Overall, quality of life was comparable between women and men. Role functioning was slightly higher in women, and emotional functioning was higher in men. Several symptom scales (especially fatigue, nausea, insomnia, appetite loss and constipation) were higher among women. Across age, there was a decline of functioning (except for emotional functioning) and global quality of life for men and women. Symptoms increased considerably over the life span (except for diarrhea and financial difficulties in men).

### Distress in melanoma survivors compared to the general population

Compared to the general population, male and female former patients reported significantly increased depression (Table 1); only female, but not male former patients reported greater anxiety.

### Quality of life of survivors compared to the general population

Table 3 shows difference scores for quality of life. Mean scores of the general population were subtracted from melanoma survivors. Compared to the general population, male and female melanoma survivors reported no difference regarding global quality of life. Melanoma survivors consistently rated their functioning lower in all specific domains. They also had higher symptom scores in almost domains (exceptions were nausea, pain and appetite loss in men).

**Table 3:** Quality of life (EORTC QLQ30): Comparisons between malignant melanoma survivors and the general population.

	Men ^1)^	Women ^1)^
**Global Quality of life**	1.1	-1.0
**Functional Scales**		
Physical	-1.9 +	-2.4**
Role	-3.4*	-2.5 +
Emotional	-5.1***	-10.8***
Cognitive	-9.2***	-9.6***
Social	-5.4***	-5.7***
**Symptom Scales**		
Fatigue	5.2***	8.8***
Nausea	.01	1.4*
Pain	-1.0	3.1*
Dyspnoea	2.7 +	5.8***
Insomnia	13.1***	18.5***
Appetite loss	-.74	1.6 +
Constipation	3.9***	7.5***
Diarrhoea	6.6***	5.6***
Financial	4.6***	2.5*

^1)^ Reported are difference scores: Mean scores of the general population were subtracted from the survivors’ scores in each age group, separately for men and for women. As older age groups were overrepresented in the melanoma sample compared to the general population, average scores for men and women, respectively, were age-adjusted by multiple regression analysis. Reported are the p-values for the group effect (melanoma vs. general population), + p<.10; * p<.05; ** p<.01; *** p<.001 (bold type).

### Determinants of distress and global quality of life

As Table 4 shows, most predictors were comparable across outcomes: Negative predictors of distress or low quality of life were low dispositional optimism and pessimism, detrimental social interactions, self-blame and fear of disease recurrence. Additional predictors were higher age (50–59; 60–69; 70+ vs. 30–39 years), lack of a partnership and siblings affected (quality of life), relatives afflicted and previous diagnosis of mental disorder (anxiety), additional somatic disease (depression) and positive reappraisal (anxiety and depression).

The results of the sensitivity analysis were similar to the results above. Only for PHQ9 (depression) there were differences in the results between both analyses for the predictors positive reappraisal and siblings afflicted, but the complete case results are more likely to be biased.

**Table 4:** Statistical predictors of depression (PHQ-9), anxiety (GAD-7) and global quality of life (QLQ30) in survivors of malignant melanoma.

	PHQ9		GAD7		Global quality of life	
	Beta ^1)^	p-value	beta	p-value	Beta	p-value
Age group of 50–59 vs. < 39 years					-7.38(-13.0 to -1.7)	.01
Age group of 60–69 vs. < 39 years					-7.95(-13.7 to -2.2)	.006
Age group of 70+ vs. < 39 years					-14.57(-20.0 to -9.2)	<.0001
partnership yes vs. no					5.16(1.3 to 9.1)	.0095
Siblings afflicted					-7.19(-13.7 to -.73)	.029
Relatives afflicted			.08(.01 to .15)	.02		
Additional disease	.07 (.00 to .13)	.045				
Mental disorder			.23(.09 to .38)	.0019		
Optimism	-.13(.08 to 1.5)	<.0001	-.11(.07 to .14)	<.0001	5.25(7.1 to 3.4)	<.0001
Pessimism	.05(-.02 to -.08)	.0013	.04(-.01 to -.17)	.015	-3.20(-5.0 to -1.4)	.0004
Detrimental interaction	.05(.00 to .09)	.044	.07(.02 to .12)	.0037	-5.17(-7.9 to -2.4)	.0002
Self-blame	.10(.02 to .18)	.016	.14(.06 to .21)	.0008	-5.87(-10.3 to -1.4)	.0094
Positive Reappraisal	.09(.05 to .14)	0.0002	.10(.05 to .15)	<.0001		
Fear of progression	.04(.02 to .05)	<.001	.03(.02 to .05)	.0002	-2.46(-3.5 to -1.5)	<.0001

^1)^ Multiple Imputation for a set of covariates selected by forward and backward selection (level of selection 5%) in single imputation data: Before analysis, scores (only PHQ9 and GAD7) were transformed according to log+1 into normal distribution in order to use a linear model; lower and upper 95% confidence interval in parentheses. Only predictors p<.05 are reported; not significant: education, residence (urban/rural), time since diagnosis, UICC stage, disease status, psychiatric treatment, ongoing medical treatment, social support

## Discussion

Unlike a previous, register-based study [11], we studied long-term follow-up, more than 8.4 years after cancer diagnosis, and, with a range of 5.7 to 12.2 years. Thus, all participants can be considered long-term survivors. We included standardized questionnaires for distress (GAD-7, PHQ-9), cancer-specific assessment of quality of life (EORTC QLQ30) and used large comparison samples from the general population regarding the primary outcome measures. In the cancer sample we also assessed a broad range of psychosocial adjustment and risk factors.

Among the former patients, mean depression and anxiety scores were higher in women compared to men. When comparing the scores across the age range, depression and anxiety was lowest in men and women aged 70+. The highest scores were found in middle adulthood (aged 50–59 years).

Global quality of life was comparable between female and male former patients. Role functioning was slightly higher in women, and emotional functioning in men. Several symptom scales (especially fatigue, nausea, insomnia, appetite loss and constipation) were higher among women. Across the age span, there was a decline of functioning (except for emotional functioning) and global quality of life for men and women. Symptoms increased considerably (except for diarrhea and financial difficulties in men).

Compared to the general population, long-term survivors of malignant melanoma reported significantly increased depression (PHQ-9), and female (but not male) survivors also reported increased generalized anxiety (albeit their mean scores were clearly below the clinical range). While global quality of life was comparable to the general population, there were consistent decrements in the specific domains of physical, role, emotional, cognitive and social functionning. Former patients also suffered more from physical symptoms, regarding fatigue, dyspnea, insomnia, constipation, diarrhea, and also from financial difficulties; women additionally suffered more from nausea, pain and appetite loss.

Former patients have obviously managed to adjust to their disease to maintain or regain a good overall quality of life. However, there remained slightly heightened (subclinical) distress, a compromised functional quality of life, physical symptoms and financial problems. Among the social variables, sex and education were not predictive of an adverse psychosocial outcome, and there was a slight benefit of the youngest vs. the oldest groups regarding overall quality of life. While distress has not consistently been associated with tumor stage in previous studies, it must be assumed that patients with progressed disease had died between diagnosis and long-term follow-up. In the survivors, confrontation with the disease in afflicted siblings or relatives played a role. Additional risk factors were physical or previous mental disease. Consistently, a negative appraisal (pessimistic stance, self-blame and fear of recurrence) were risk factors for heightened distress and reduced quality of life. Consistent with findings in long-term surviving breast cancer patients, detrimental social interactions such as disappointments, lack of consideration, understanding and support may have an additional negative impact [24]. It may seem surprising that positive reappraisal turned out as a risk factor; however, more need for coping measures may also indicate heightened distress.

Overall, our findings support implementation of regular psychooncological screening as routinely recommended over the aftercare period of 10 years in the current German medical guidelines in order to identify and support those patients who have difficulties adjusting to their disease in the long run, but are still below the threshold of substantial mental disorder [25]. Screening should include distress and specific aspects of quality of life (functional and symptom scales). The interplay of vulnerability factors (e.g. previous mental disease), confrontation with the threat of cancer (e.g. in the family), appraisal, coping and social support may determine long-term adjustment and indicate specific needs for information and psychooncological support. Thus, prospective study is needed to determine the course of adaptation to the disease and corroborate the risk factors identified.

### Limitations

While the register-based recruitment has the advantage [10] that we can assume that more than 95% of melanoma patients have been registered in the state-wide registry, protection of confidentiality required indirect recruitment. As we had to rely on the cooperation of the reporting physicians as many other European trials, attrition was slightly higher (48% of contactable patients) than in other registry-based studies allowing direct contact to former patients [10]. Also, the long follow-up period has probably added to the attrition. Thus, we cannot be sure if we overestimated or underestimated the level of distress and quality of life in the melanoma survivors. We performed direct comparisons with large and representative surveys of the German population. Depression and quality of life were assessed in the general population about the same time as the patient survey was performed; anxiety data came from a survey performed 6 years earlier. Sampling procedures and participation rates were identical; however, we could not determine the proportions of cancer and other chronic diseases in the general population. Different age distributions between the melanoma and the population sample were taken into account statistically. Another limitation pertains to missing data for tumor staging; however, given the long-term course, survival of patients with progressed disease could not be expected in many cases of our sample.
